# Boots for Belgian Wounded : Thanks from Worcester

**Published:** 1915-01-16

**Authors:** M. Herbert

**Affiliations:** Matron, General Infirmary, Worcester.


### THE EDITOR'S LETTER-BOX.

### Boots for Belgian Wounded: Thanks from Worcester.

*To the Editor of* THE HOSPITAL.

SIR,—Allow me to thank you most sincerely on behalf of the four Belgian soldiers for the splendid boots with which you have provided them. They will be most acceptable now, enabling them to take good walks, in spite of rain, in the county to which they have gone for their convalescence.—Faithfully yours,

M. HERBERT, Matron,
General Infirmary, Worcester.

